# Heat-shock proteins in infection-mediated inflammation-induced tumorigenesis

**DOI:** 10.1186/1756-8722-2-5

**Published:** 2009-01-30

**Authors:** Mark G Goldstein, Zihai Li

**Affiliations:** 1 University of Connecticut, 263 Farmington Avenue, Farmington, CT 06030, USA; 2 Center for Immunotherapy of Cancer and Infectious Diseases, University of Connecticut Health Center, 263 Farmington Avenue, Farmington, CT 06030, USA

## Abstract

Inflammation is a necessary albeit insufficient component of tumorigenesis in some cancers. Infectious agents directly implicated in tumorigenesis have been shown to induce inflammation. This process involves both the innate and adaptive components of the immune system which contribute to tumor angiogenesis, tumor tolerance and metastatic properties of neoplasms. Recently, heat-shock proteins have been identified as mediators of this inflammatory process and thus may provide a link between infection-mediated inflammation and subsequent cancer development. In this review, the role of heat-shock proteins in infection-induced inflammation and carcinogenesis will be discussed.

## Introduction

Since the time of Rudolf Ludwig Karl Virchow, inflammation has been implicated as a necessary albeit insufficient component in tumorigenesis in some cancers [1,2]. Recent research has characterized several molecular mechanisms that demonstrate such a link. In addition, numerous infectious agents have been directly implicated as the source of this inflammatory pathway. Studies have shown that the innate and adaptive immune systems that respond to these infections may be directly responsible for tumor angiogenesis, tumor tolerance and in some cases metastatic mechanisms by providing the tumor with cytokines that promote these processes. One of the more recent discoveries has been the role of heat-shock proteins as mediators of this immune-mediated process via tumor peptide presentation [3]. In this review, we will discuss briefly the anti-cancer properties of heat-shock proteins and emphasize their critical faculties in infection-mediated inflammation-dependent tumorigenesis.

An estimated 10.9 million new cases of cancer occurred in 2002 worldwide. In 1990 investigators at the International Agency for Research on Cancer estimated that approximately 9% of cancers in the United States and 20% of cancers in developing countries could be attributed to infectious agents [4]. This geographic disparity may be due to the higher prevalence of cancer-related infectious agents in developing countries [5]. Cancers caused by such infections theoretically occur as a result of direct cell targeting with subsequent tumor suppressor gene inactivation, as in human papilloma virus (HPV), prolonged local inflammation by bacteria residing outside of tumor cells, such as H. *pylori*, or immune suppression by viral agents, such as human immunodeficiency virus [6-8]. Conversely, in the 1700s cancer patients who cleared bacterial infections occasionally experienced remission of their established malignancies [9]. In the late 1800s, Dr. William B. Coley of the New York Cancer Center noted the regression of sarcoma in patients who developed erysipelas [10]. Despite these isolated findings, the preponderance of evidence shows that infections contribute to carcinogenesis rather than counter it. A comprehensive explanation of this relationship has yet to be described.

### Inflammation, tumor immunity and tumorigenesis

Inflammation is a localized protective response elicited by injury or destruction of tissues which serves to destroy, dilute or wall off both the injurious agent and the injured tissue. The inflammatory response to infections as well as other stimuli involves a myriad of defenses, including both the innate and adaptive arms of the immune system.

The innate immune system is comprised of myeloid cells such as macrophages and dendritic cells, and innate lymphocytes such as natural killer cells, all of which lack immunologic memory. This cellular component of the innate immune system can either kill engulfed microbes using toxins including superoxide anion, hydroxyl radical and nitric oxide or process antigens in a MHC-dependent manner. Extracellular antigens such as bacterial toxins are presented by MHC class II on antigen presenting cells (APCs) to CD4+ T cells whereas intracellular antigens such as viral antigens are presented by MHC class I to CD8+ T cells [11]. These APCs are stimulated by germline-encoded innate receptors such as Toll-like receptors (TLRs) to program adaptive immunity (both cellular and humoral immunity) via cytokines, co-stimulatory molecules in addition to present antigens to T cells [12].

The immune system therefore can function to modulate tumorigenic pathogen-induced chronic inflammatory responses or to identify and eliminate tumor cells. The latter process now known as *immunologic tumor surveillance *was first proposed by Burnet in 1957 [13]. When these events result in tumor clearance, it is known as *elimination*. If not cleared, a state of *equilibrium *between the tumor-suppressive immune system and tumor growth can occur. If tumor *immunoediting *progresses, the tumor grows or *escapes *[14,15]. Tumor immunologists in the past several decades have been focusing on the immune system to counter cancer. Increasing evidence is uncovering the paradoxical roles of the immune system to promote tumorigenesis.

The ancient Roman physician Galen (129 – 199 C.E.) was the first to posit the causal relationship between cancer and inflammation. In 1863, the "Father of Pathology," Rudolf Virchow perpetuated the notion that cancers must be due to prolonged irritation of various sorts. Similarly, Dr. C. Heitzman declared in 1883 that the "so-called small cellular infiltration [of Virchow] of the connective tissue was the 'pre-stage of cancer"' [16]. Since that time, the study of inflammation has become increasingly complicated, albeit more cohesive, in its associations with cancer [17]. Ultimately, chronic inflammation has been shown to contribute to tumorigenesis by causing DNA damage, promoting neoangiogenesis and compromising tumor immunosurveillance mechanisms.

**Figure 1:**
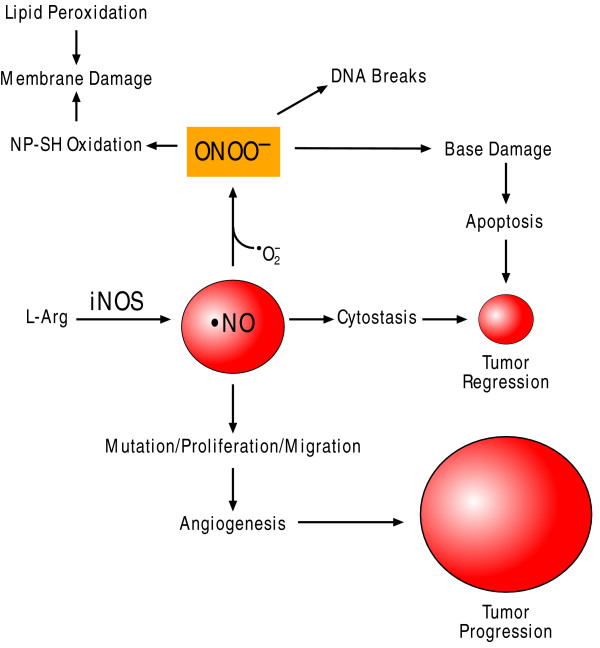
**Growth and inhibitory effects of free radicals on tumors**. The unchecked production of hydroxyl radicals and other reactive oxygen species (ROS) leads to protein and lipid peroxidation as well as DNA damage which increase mutation load resulting in either tumor regression or tumor progression. In response to intracellular protozoa, classically-activated macrophages produce nitric oxide (NO) from arginine (L-arg) using the iNOS enzyme. H.*pylori *disinhibits iNOS in the gastric mucosa by attenuating the expression of HSP70 and HSP27. Tumor-associated macrophages (TAM) are not toxic to tumor cells because of their limited production of NO.

Free radicals are thought to mediate tumorigenesis in the context of inflammation. Excess oxidative/nitrosative stress results in the generation of reactive oxygen species (ROS) such as hydroxyl radicals (OH·) and ultimately the accumulation of protein peroxidation, DNA damage and lipid peroxidation (LPO) (Figure 1) [18]. ROS and reactive nitrogen species (RNS) can damage both nuclear and mitochondrial DNA, RNA, lipids and proteins by nitration, oxidation and halogenation reactions, leading to an increased mutation load [19]. The LPO products [*trans*-4-hydroxy-2-nonenal (HNE), 4-hydroperoxy-2-nonenal (HPNE), and malondialdehyde (MDA)] can drift far from membranes and cause exocyclic adducts on DNA that are potentially promutagenic if not removed [20].

In human lung bronchial epithelial cells, the proinflammatory cytokine TNF-α has been shown to induce production of such ROS with a concomitant increase in 8-oxo-deoxyguanosine, a marker for oxidative DNA damage. The source of the ROS was shown to be spermine oxidase [21]. In vivo humans and experimental animals have been found to harbor carcinogenic N-nitrosamines formed by the deamination of DNA bases by N2O3 [22].

In the case of colon cancer, commensal intestinal flora can activate TLRs on the luminal surface of intestinal epithelial cells [23]. This interaction activates intracellular IKK-β and ultimately NF-κB, the key regulator of inflammation found in many solid tumors [24]. NF-κB is a homo- or hetero-dimeric transcription factor of the Rel family. NF-κB activates genes involved in cell proliferation (e.g., c-myc, cyclins), as well as cell survival (e.g., c-FLIP, c-IAP1, c-IAP2, XIAP, Bcl-XL, Bfl-1/A1 and p53) [25]. NF-κB contributes unevenly to the pro-apoptotic and anti-apoptotic pathways dependent upon its role in homeostasis or tumor development, respectively [26]. In a pro-inflammatory state, NF-κB contributes to the activation of COX-2, iNOS and matrix metalloproteinase (MMP-9). Furthermore, NF-κB is responsible for the expression of adhesion molecules and cell-surface metalloproteases, including MMP-9 and MMP-2, substances which degrade the extracellular matrix (ECM) to allow for metastases [27,28]. Downstream of NF-κB, increased expression of pro-inflammatory COX-2 has been demonstrated in colorectal adenomatous polyps and has been linked to the induction of tumorigenic DNA damage [18].

The tumor microenvironment features an important inflammatory cell component as well. Currently, it is believed that there are three types of activated macrophages. The classically activated macrophage which responds to intracellular pathogens is stimulated by IFN-γ, stimulates T-cells with IL-12, and produces nitric oxide (NO) from arginine using the iNOS2 enzyme (Figure 1). The so-called alternatively activated macrophages are stimulated by IL-4, fail to make NO, and inhibit T cell proliferation, but are able to produce IL-1-receptor antagonist and IL-10. The type 2-activated macrophages induce T_H_2-type humoral immune responses to antigen, such as IL-10 generation which results in IL-4 production by T cells, and leads to an anti-inflammatory milieu [29].

One key inflammatory component to tumor sustenance first discovered in the late 1970s is the infiltration of tumor-associated macrophages (TAM) which are attracted by monocyte chemotactic protein (MCP-1), RANTES and CCL5. TAMs accumulate in poorly vascularized and relatively hypoxic zones of tumor where hypoxia-inducible factors (HIF-1 and HIF-2) predominate and promote expression of pro-angiogenic VEGF, bFGF, and CXCL8 [30-34]. Like type 2-activated macrophages, TAMs release IL-10, PGE-2, TGF-β and other cytokines that inhibit antigen presentation and normal DC activity [35]. They are not cytotoxic for tumor cells because of their limited production of NO and proinflammatory cytokines and due to the production of IL-10 which dampens cytotoxic T-cell reactivity [36,37]. The Sea squirt-derived trabectidin has a selective cytotoxic effect on TAMs by binding to the minor groove of DNA and reducing IL-6 production, resulting in tumor growth suppression [38].

When functioning in concert, these processes may prevent adequate immunosurveillance. As proof of principle, Luo et al demonstrated that a legumain-based DNA vaccine induced a robust CD8+ T cell response against TAMs, dramatically reducing their presence in tumor tissues and decreasing proangiogenic TGF-β, TNF-α, MMP-9 and VEGF. Subsequently, tumor angiogenesis, tumor growth and metastases were suppressed [39].

### Heat-shock proteins

First discovered accidentally in 1962 by Ritossa et al and isolated in 1974 by Tissieres et al, heat-shock proteins (HSPs) are a highly conserved group of protein products generated as a result of natural stressors, such as fever and active commensal gut microflora, or non-natural stressors, such as hyperthermia, NSAIDS, aspirin, nutrient withdrawal, ROS, proteasome inhibition, UV radiation and chemotherapy-induced DNA damage [40,41]. They promote cell survival by preventing mitochondrial outer membrane permeabilization, cytochrome *c *release, caspase activation and apoptosome assembly [42]. HSPs assist in general protein folding to prevent non-specific aggregation of misfolded or unfolded proteins which would otherwise be rendered nonfunctional. This folding process is facilitated by cofactors such as Hsp70/Hsp90 Organizing Protein (HOP) which associates with Hsp70 and Hsp90 to mediate the transfer of polypeptides from Hsp70 to Hsp90. Conversely, Hsp70 and Hsp90 may associate with the ubiquitin ligase CHIP and lead to proteasomal degradation of a misfolded protein.

Highly inducible HSPs such as HSP70 and HSP27 are transcriptionally controlled by heat shock transcription factor trimers, such as *hsf*1. For example, *hsf*1 represses transcription when bound to HSP70 during attenuation of the heat shock response as a negative feedback mechanism [43]. In a normal host, *hsf1 *enhances organismal survival and longevity. In cancer, however, *hsf*1 in particular has been found to be overexpressed and to contribute to invasion and metastasis by permitting increased cell proliferation and by decreasing cell death [44-47]. As expected, genetic deletion of *hsf*1 protects mice from experimental tumors [48].

HSP activation can directly affect both innate and adaptive immunity, although controversial studies and opinions exist in the field [49-51]. The innate immune responses induced by HSPs include cytokine and chemokine release by professional APCs and T-cells, maturation of DCs by upregulating the expression of costimulatory and antigen-presenting molecules such as B7-1, B7-2 and MHC-II molecules, induction of migration of DC to draining lymph nodes and activation of NK cells [52].

**Figure 2:**
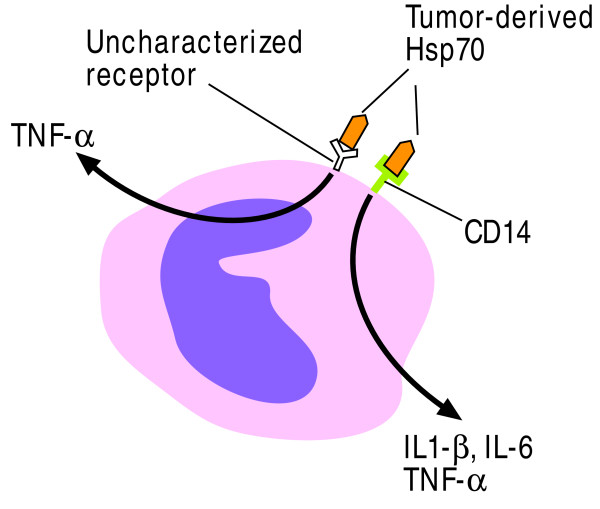
**Heat-shock protein signal cascade**. Necrotic tumor-derived mammalian gp96 and HSP70 can signal antigen-presenting cells (APCs) via CD14, and other receptors such as TLRs and CD91 which remain to be fully determined.

For example the HSP gp96 interacts with TLR2/4 resulting in the activation of NF-κB-driven reporter genes and mitogen- and stress-activated protein kinases. Gp96 also induces the degradation of IκBα in DCs while simultaneously stimulating both the innate and adaptive immune system [53]. Gp96-activated DCs release proinflammatory cytokines resulting in the induction of an inflammatory response by the innate component of the immune system [54]. Necrotic tumor cell-derived mammalian gp96 and hsp70 signal APCs via CD14, TLRs and CD91 (Figure 2) [55-58]. Tumor-derived Hsp70 can also activate NK cells having a high cell surface density of CD94 [59] by inducing NKG2D ligands on the surface of DCs [60]. This scenario may be particularly relevant in melanoma which overexpresses Hsp70.

The immunogenic potential of gp96-peptide complexes was first demonstrated by Srivastava et al [61,62]. When manipulated, tumor-derived gp96 vaccine induces T cell priming and tumor rejection [63-65]. When HSP-peptide complexes are procured by APCs, peptide is transferred from HSPs to MHC molecules for recognition by T cells [61]. Dai et al found that cell surface expression of gp96 leads to the priming and maintenance of both CD4+ and CD8+ T cell immunity against tumors and potentiates cross-presentation of intracellular antigens to MHC-I for activation of CD8+ T cells [66]. The interaction of gp96 with DCs leads to the preferential expansion of antigen-specific CD8+ T cells in vitro and in vivo in a TLR4-dependent manner [67]. These CD8+ T cells can then contribute to tumor immunosurveillance.

Furthermore, HSPs have been shown to induce T cell regulation of chronic inflammation [68]. HSPs can chaperone both steroid and non-steroid hormone receptors. Interestingly, steroids can interact with HSP-bound glucocorticoid receptors and increase the expression of IκBα, preventing the nuclear translocation of the pro-inflammatory molecule NF-κB [69,70].

### Heat-shock proteins and tumorigenesis

**Figure 3:**
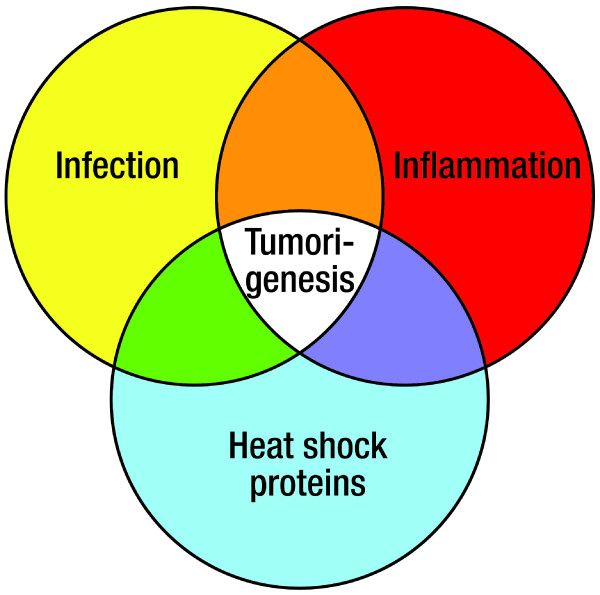
**Venn diagram demonstrating a model of the tumorigenic relationship between infection, chronic inflammation and microbial- or host-derived heat-shock proteins**.

The histologic evidence of chronic inflammation resulting from an infection is insufficient to explain a tumorigenic mechanism. This shortcoming can partially be reconciled by the identification of HSPs in and around tumors (Figure 3). Heat-shock proteins can be produced by tumors, microbes, and even inflammatory cells in the tumor microenvironment. Only recently have HSPs been implicated as biochemical elements of both anti-tumor immunity [3] and oncogenesis [48].

Unique HSPs activated in cancer cells have been well-documented and correlated with tumor cell proliferation, differentiation, invasion, metastasis and prognosis. Frequently, the tumor-derived HSPs are acetylated [71] and cannot be directly compared with native HSP or microbial HSP. Gp96 from tumor cells demonstrate greatly altered glycosylation patterns compared to host cell gp96, which may elucidate deficiencies in immune surveillance [72]. Tumor-derived Hsp90 can rescue wild type proteins as well as unstable mutant proteins implicated in carcinogenesis. Moreover, tumor-derived HSP90 is present entirely in multi-chaperone complexes with high ATPase activity, unlike non-tumor HSP90 [73,74]. For example, in chronic lymphocytic leukemia (CLL), ZAP-70+ lymphocytes express activated HSP90 which binds and stabilizes ZAP-70 with several HSP co-chaperones [75].

The HSP90 family consists of cytoplasmic HSP90β, inducible α-form, GRP94/gp96 and mitochondrial TRAP1/hsp75. HSP90 is a constitutively active, molecular chaperone that assists in folding of signature tumorigenic proteins such as HER-2/ErbB2, Akt, Raf-1, v-Src, and Bcr-Abl [76]. HSP90 is overexpressed in a wide variety of solid and hematologic malignancies and correlates with a poor prognosis [77]. The expression of endoplasmic reticulum regulator HSP70-member GRP78 (also known as BiP), glucose-regulated protein GRP94/gp96, or HSP90 has been associated significantly with vascular invasion and intrahepatic metastasis [78]. HSP90 may even promote invasion of metastases by chaperoning NF-kB-dependent MMP-2 [79].

Cultured cells and transgenic mice have been shown to exhibit cellular transformation and tumor formation when forced to over express intracellular HSP27 or HSP70 [80-83]. It has even been proposed that by interacting with mutant p53 and various oncogene products such as pp60-v-src, fes and fgr, these HSPs may alter cell cycle regulation and contribute to the anti-apoptotic mechanism of tumorigenesis [84].

HSP70 belongs to a family of inducible chaperone proteins frequently present on the plasma membrane of colon, lung, pancreas and breast cancer metastases [85]. This ATP-dependent chaperone can be induced by a variety of stimuli, including chemotherapy. HSP70 is a powerful anti-apoptotic protein that reduces caspase activation and suppresses mitochondrial damage and nuclear fragmentation [86]. HSP70 can even subvert apoptosis by blocking the translocation of Bax, which results in stabilization of the outer mitochondrial membrane [87]. HSP70 is also a potent activator of the human complement system in an antibody-independent fashion [88]. In defense, cancer cells block complement-mediated killing by expressing membrane complement regulatory proteins, such as CD46, CD55, CD35 and CD59 [89].

HSP27 of the inducible small HSP family has been shown to inhibit the mitochondrial release of SMAC (second mitochondrial-derived activator of caspase), the master regulator of apoptosis, to confer resistance of multiple myeloma cells to dexamethasone [90]. Conford et al have found a high correlation between the level of HSP27 expression and the Gleason score in prostate cancer [91]. HSP40, HSP60, and HSP70 expressions are up-regulated in response to the development of high grade intraepithelial neoplasia and cervical cancer [92]. These examples begin to unveil the complex relationship between HSPs and cancer formation.

### Microbes, inflammation, heat-shock proteins and cancer

The parasitic origin of cancer was originally suggested by Paget in 1887 [93].

"I believe that microbe parasites, or substances produced by them, will some day be found in essential relation with cancer and cancerous disease."

In 1913, Dr. Johannes Fibiger, the pathological anatomist in Copenhagen, produced numerous cancers in the fore-stomach of rats by feeding them a nematode taken from the muscles of a cockroach [94]. Similarly, Bullock and Curtis produced hepatic sarcomas in rats by feeding them tapeworm eggs from cats [95]. And Schistosoma, a parasitic trematode or fluke discovered in 1851 by Theodor Bilharz, has been shown to cause chronic local inflammation which seems to increase the risk of developing squamous cell bladder cancer [96]. Over 200 million people in tropical and subtropical countries are believed infected by any of six species of schistosomes. In Egypt alone, 27% of the 2500 new cancer patients each year have bladder cancers attributed to schistosomiasis [97].

Adult schistosome trematodes are found in the venous plexus around the urinary bladder. Any eggs released can then traverse the bladder wall and cause hematuria. Immune responses during the early stages of schistosomiasis infection are directed against antigens of schistosomula, and demonstrate a T_H_1 profile. With the onset of egg laying, T_H_1 responses are replaced by vigorous T_H_2 responses directed against egg antigens. The result is a tissue granuloma surrounding eggs characterized by an infiltrate of T_H_2 cells, eosinophils, macrophages and fibroblasts within a dense collagen-rich matrix. Schistosome-induced macrophages and neutrophils are important sources of endogenous oxygen or hydroxyl radicals, which are also implicated in the formation of carcinogenic *N*-nitrosamines [98]. These inflammatory cells may induce genotoxic effects, such as mutations, sister chromatid exchanges and DNA strand breaks [99-101]. They may also participate in the activation of procarcinogens, such as aromatic amines and polycyclic aromatic hydrocarbons, generating carcinogenic metabolites [102]. An increased number of inflammatory cells in the urinary bladder of schistosomal patients may enhance the carcinogenic potential of these agents by increasing their rate of activation. Furthermore, in patients with S. *haematobium *and bladder cancer, TAMs attracted to the bladder can produce TNF alpha, a key component of inflammation which is upregulated by HSP60 and HSP90.

HSP can be produced by a wide variety of parasitic organisms as detailed by Maresca et al [103]. HSP86, HSP70, HSP60, HSP58, HSP27 have all been detected in S. *mansoni*. In superficial transitional cell bladder cancer, the loss of surface expression of tumor-derived HSP60 and HSP90 was correlated with a poor prognosis, possibly explained by the inability of T cells and NK cells to recognize these tumor cells [104]. In the future these findings may champion parasite-derived HSPs as potential carcinogens.

Bacteria have also been implicated as a cause of cancer. In 1893, Bizzozero discovered a spirochete in the stomach of dogs. This finding has since been verified by numerous scientists including Salomon in 1896 and Krienitz in 1906 who related a similar finding to gastric cancer in a human patient [105]. More importantly, in 1983 the microbe now known as Helicobacter *pylori *was identified as a trigger of gastric cancer and gastric lymphoma [106-108]. H. *pylori *is associated with infiltration by neutrophils and mononuclear cells in gastric mucosa, likely attracted by granulocyte macrophage colony-stimulating factor and RANTES. Subsequently macrophages and monocytes respond to the presence of H. *pylori *via TLR2 resulting in NF-κB activation and the release of early proinflammatory cytokines, such as IL-1β. Macrophage-derived migration inhibitory factor (MIF) is a potent cytokine produced by H. *pylori *that overrides tumor suppressor p53 activity by suppressing its transcriptional activity. The result is increased DNA damage by inflammatory cells [109]. Furthermore, H. *pylori *infection disinhibits iNOS (Figure 1) in the presence of lipopolysaccharide by significantly attenuating the expression of HSP70 and HSP27 [110]. As expected, increased iNOS expression and subsequent oxidative damage has been found in gastric mucosa chronically infected with H. *pylori *[111].

Corresponding increases in various cytokines including IL-1β, IL-6, IL-8, and TNF-α have also been identified [112]. Investigators have shown that H. *pylori *must directly contact the host cell in order to up-regulate IL-8 [113]. NF-κB-dependent expression of IL-8 has been correlated with increased vascularity in human gastric carcinomas [114]. Takenaka et al have demonstrated how H. *pylori*-derived HSP60 can activate NF-κB and mitogen-activated protein kinase (MAPK) and induce IL-8 production and secretion through TLR-2 and TLR-4 pathways in KATO III human gastric epithelial cells [115,116]. HSP62, a member of the HSP60 chaperonin family and homologue of the H. *pylori *HSP known as GroEL, has been shown to participate in the extracellular assembly of H. *pylori *-derived urease, a known virulence factor [117]. These mechanisms provide insight into the relationship between H. *pylori *infection, inflammation, HSPs and tumorigenesis.

Chlamydial HSP60 has also been recognized as a potential extracellular stimulus of oncogenesis in that it is found in pre-neoplastic lesions and can bind TLRs, inducing a cascade of signaling which leads to neoangiogenesis, macrophage activation and anti-apoptosis mediated by complexing with Bax and Bak [118]. However, there is limited evidence which can implicate microbial or host HSPs as directly carcinogenic.

Parasites and bacteria are not the only culprits. In 1911, Dr. Peyton Rous of the Rockefeller Institute first demonstrated the RNA retrovirus causally associated with sarcomas in chickens for which he received the Nobel Prize in 1966 [119]. Since then several human cancers have been attributed to viral infections although the exact mechanism has not been elucidated in every case.

In 1963, Blumberg discovered the Hepatitis B virus (HBV), which is now known to cause hepatocellular carcinoma (HCC) in humans. Cell surface expression of viral HBsAg and HBcAg in association with MHC class I molecules activates CD8+ cytotoxic T lymphocytes which can then produce IFN-gamma. Hepatic GRP94/gp96, an endoplasmic reticulum-associated member of the HSP90 family, has been observed in association with HBV DNA and core antigen protein in biopsies of HCC [120,121]. Hepatic gp96 expression has been correlated with the degree of tumor differentiation and tumor size [120]. The exact role of gp96 in this case has not been determined. Interestingly, expression of the SMAC-inhibitor HSP27 has been shown to correlate with prognosis, disease-free and overall survival in patients with HBV-associated HCC [122].

The Epstein-Barr virus (EBV) is highly prevalent in humans (≥ 90% worldwide are carriers). In 1964, Epstein described EBV in association with endemic Burkitt's lymphoma in Central Africa, a highly aggressive but potentially curable form of non-Hodgkin lymphoma, as well as nasopharyngeal carcinoma. EBV is able to bind CD21 on B cells, a critical event to the induction of HSPs and the transformation of some B cells enabling them to become independent of the usual regulatory factors, including T cells. Cheung et al described in detail the coordinate induction of HSP70 and HSP90 at mRNA and protein levels upon EBV infection in vitro. Induction of HSPs and transformation of B cells were dependent on EBV-induced *trans*-membrane Ca2+ currents, but not on EBV gene products. Blockade of HSP induction prevented transformation [123]. This evidence has been essential for deciphering the role of HSPs in tumorigenesis.

## Conclusion

For over two millennia scientists have speculated the etiology of cancer. In some instances such as tobacco use, there is a preponderance of evidence demonstrating a direct carcinogenic link with tobacco use. The roles of chronic infections and chronic inflammation have been repeatedly investigated as tumorigens for over a century with only a handful of confirmed associations relative to the diversity of human neoplasms and pathogens. Nevertheless, the worldwide population burden of infectious organisms makes understanding their role in human disease of paramount importance to cancer prevention strategies. Molecular studies have been able to dissect the pathophysiology of carcinogenesis on many levels. The direct and indirect involvement of microbial or host heat-shock proteins in the malignant transformation of a chronically infected host has been shown to be integral. This review attempts to assemble the evidence implicating heat-shock proteins in the neoplastic process.

Ever since the crucial role of heat shock proteins in cancer pathophysiology was established, efforts to inhibit their carcinogenic capacity have taken many forms. Heat-shock protein vaccines using conjugated tumor peptides [124,125] and direct HSP90 inhibitors such as 17-(Allylamino)-17-demethoxygeldanamycin (17-AAG) [76] have been investigated in clinical trials. Currently these interventions have not proven efficacy clinically, although they seem promising in vitro and in early phase trials. It remains to be seen whether or not manipulation of one HSP at a time will lead to meaningful tumor responses and/or survival benefit.

## Competing interests

The authors declare that they have no competing interests.

## Authors' contributions

All authors, MG and ZL, participated in drafting and editing the manuscript. MG and ZL read and approved the final manuscript. The cited work from the laboratory of ZL was supported by grants from NIH, DHHS, USA.

## Authors' information

The authors provided specialized, multidisciplinary clinical care for hematology and oncology patients at the University of Connecticut Neag Comprehensive Cancer Center – John Dempsey Hospital. ZL is currently an Associate Professor in the Department of Immunology at the Univesity of Connecticut and a clinical scholar of the Leukemia and Lymphoma Society, USA. MG has completed fellowship training at the University of Connecticut and is currently in private practice in Maryland.
